# The echo from outside: ASCORBATE PEROXIDASE 1 modulates cytosolic effector-triggered reactive oxygen species

**DOI:** 10.1093/plphys/kiad089

**Published:** 2023-02-15

**Authors:** José Manuel Ugalde

**Affiliations:** Assistant Features Editor, Plant Physiology, American Society of Plant Biologists, Rockville, USA; INRES—Chemical Signalling, University of Bonn, Friedrich-Ebert-Allee 144, 53113 Bonn, Germany

Plants and pathogens are in a never-ending battle that has pushed plants to develop a complex immune system to endure pathogen attacks. Such a system is initially triggered when the plant senses an incoming pathogen, like bacteria, by recognizing molecular patterns (PAMPs) present on the pathogen surface, activating PAMP-triggered immunity (PTI) in the plant. Bacteria have evolved effector proteins secreted into the host cell to suppress the immune response. In turn, plants have developed receptors for these effectors that, upon recognition, trigger an effector-triggered immunity (ETI) (Jones and Dangl 2006).

**Figure 1.**
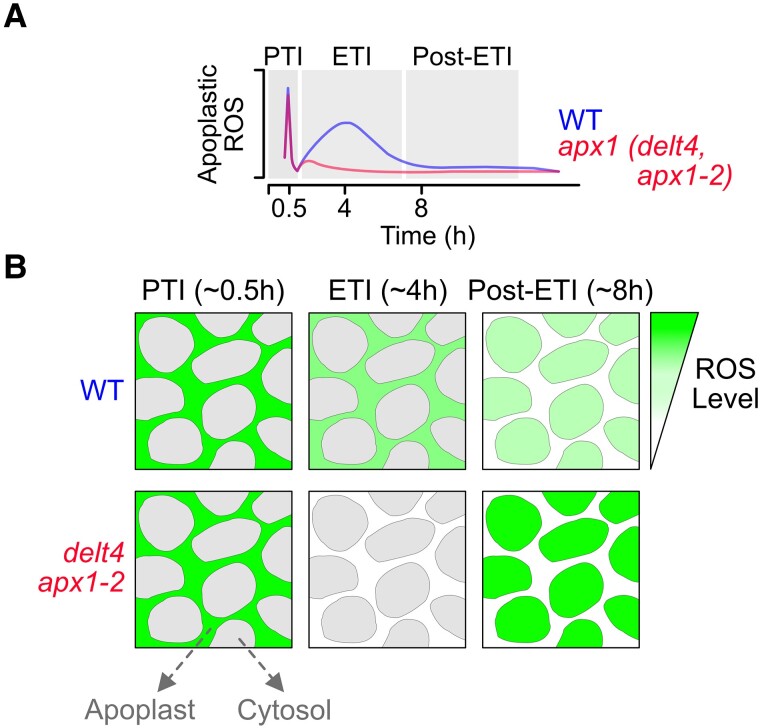
ROS levels in response to biotic stress. **A)** Scheme indicating the apoplastic ROS levels as detected with the luminol chemiluminescent assay in WT plants and mutants deficient in *APX1* (*defective in LPS-triggered ROS production 4*, *delt4* and *apx1-2*). Upon infection or treatment with LPS, a transient ROS burst will be detected as early as 0.5 h from the PTI. A second ROS burst from theETI will be detected with a peak around 4–5 h. The second peak is almost not detected in the *apx1* mutant lines. **B)** Scheme indicating the ROS levels of the apoplast and cytosol of WT and *apx1* mutant cells infected with an avirulent bacteria. In the time points following ETI, there is an increase in the cytosolic ROS that is consistently larger in the *apx1* mutants compared to WT. (Figure created by J.M.U. in Affinity Designer, Version 2.0.0).

Both PTI and ETI signals have the capacity to trigger calcium influx, the activation of protein kinases, and the production of reactive oxygen species (ROS) in the apoplastic region via membrane-bound NADPH oxidases (RBOHs) (Torres et al. 2002). RBOHs generate superoxide that will spontaneously dismutate into the more stable hydrogen peroxide. Chemiluminescent assays with luminol have contributed to establishing how hydrogen peroxide levels in the apoplast increase biphasically after infection with an avirulent pathogen and, more recently, by exposure to lipopolysaccharide (LPS), a bacterial PAMP (Yuan et al. 2021; Wu et al. 2022). ROS production induced by PTI occurs 0.5–1 h after infection, with a fast and short burst, while ETI-induced ROS production has a slower and prolonged increment that peaks 4–8 h after infection (Fig. 1A). Apoplastic ROS constitute a crucial part of the signaling pathway that informs the cell of an incoming attack. Hence, ROS produced in the apoplast needs to enter the cytosol. The transport of ROS is facilitated by aquaporins that also serve as channels for water (Bienert et al. 2007). Nonetheless, there remains a substantial gap in our knowledge of how these ETI-induced ROS signals reach the cytosol and how they are modulated.

Recently in *Plant Physiology*, Hong et al. (2022) searched for *defective in LPS-triggered ROS production* (*delt*) mutants from a collection of mutagenized Arabidopsis (*Arabidopsis thaliana*) plants. The authors isolated a *delt4* mutant and confirmed it to be deficient in cytosolic *ASCORBATE PEROXIDASE 1* (*APX1*). Their research demonstrated how sustained apoplastic ETI-ROS triggered by pathogen infections or LPS treatments is eventually detected in the cytosol and that this process depends on the activity of APX1.

In their screening, the authors measured LPS-induced ROS in the apoplast of the *delt4* mutant and showed almost no ETI-induced ROS production occurred after LPS treatments in this line compared to wild-type (WT) plants (Fig. 1). They confirmed that a knockout line for *APX1 (apx1-2)* is also deficient in triggering an ETI response, observed as a lack of the second LPS-induced ROS burst (Fig. 1A). The authors verified that the limited ETI response also occurs in APX1 deficient lines infected with the avirulent pathogen *Pseudomonas syringae* (*avrRpt2*). The authors found no difference between the *apx* mutants and WT in the expression levels of genes linked to other antioxidant systems, nor changes in the protein levels or activity of RBOHD, the most relevant RBOH in the onset of the immunity response. These results indicate that the limited apoplastic ROS production in the *apx* mutants is not due to a compensatory effect of other antioxidant system components or deficient RBOHD-mediated ROS production. Using ROS dyes such as H_2_DCFDA and Aurafin, they measured LPS-induced ROS levels in the apoplast and the cytosol and confirmed incremental increases in cytosolic ROS levels following ETI that were higher in the lines deficient in APX1 (Fig. 1B).

The data presented reveal a putative role of cytosolic APX1 in the regulation of apoplastic and cytosolic ETI-induced ROS under biotic stress. Such discovery constitutes evidence of how ROS produced during the ETI response eventually increases the cytosolic ROS levels. Further work is required to understand how this regulation occurs and to determine if all the cytosolic ROS measured by the dyes comes from the apoplast or if intracellular ROS production is activated by either part of this extracellular ROS, calcium, or other signaling molecules.

ROS dyes and the chemiluminescent assay have proven to be highly efficient platforms to analyze mutant collections. Yet, they are restricted in their subcellular resolution and mostly limited to depicting the cumulative ROS generated during a time window rather that the ROS dynamics over time. Genetically encoded biosensors targeted to either the apoplast or cytosol (Arnaud et al. 2023), able to sense H_2_O_2_, such as roGFP2-Orp1 (Nietzel et al. 2019), or the highly sensitive HyPer7 (Ugalde et al. 2021) expressed in *apx1* mutants or lines deficient in aquaporins would provide a real-time view of ROS transit during pathogen infections.
